# Patient-caregiver agreement and test–retest reliability of the EQ-5D-Y-3L and EQ-5D-Y-5L in paediatric patients with haematological malignancies

**DOI:** 10.1007/s10198-021-01309-w

**Published:** 2021-05-05

**Authors:** Wenjing Zhou, Anle Shen, Zhihao Yang, Pei Wang, Bin Wu, Michael Herdman, Nan Luo

**Affiliations:** 1 Department of Paediatrics, Renji Hospital, School of Medicine, Shanghai Jiaotong University, Shanghai, China; 2 Medical Psychology and Psychotherapy, Erasmus University, Rotterdam, The Netherlands; 3 Department of Pharmacy, Shanghai Children’s Medical Centre, School of Medicine, Shanghai Jiaotong University, Shanghai, China; 4 College of Pharmacy, Jinan University, Guangzhou, China; 5 School of Public Health, Fudan University, Shanghai, China; 6 Medical Decision and Economic Group, Department of Pharmacy, Renji Hospital, School of Medicine, Shanghai Jiaotong University, Shanghai, China; 7 Office of Health Economics, London, UK; 8 Saw Swee Hock School of Public Health, National University of Singapore, 12 Science Drive 2, Singapore, Singapore

**Keywords:** EQ-5D-Y, Proxy, Reliability, Haematological malignancies, I19

## Abstract

**Background:**

In 2019, the EuroQol Group developed a ‘beta’ 5-level version of EQ-5D-Y (Y-5L) by increasing the number of descriptive levels to five for each health dimension, as compared to the standard 3-level EQ-5D-Y (Y-3L).

**Objective:**

To assess patient-caregiver agreement and test–retest reliability of the Y-5L and Y-3L in paediatric patients with haematological malignancies.

**Methods:**

Paediatric inpatients aged 8–17 years were interviewed with the Y-5L and Y-3L questionnaires twice, while their caregivers were interviewed at the same time using the proxy versions of the questionnaires. Patient-caregiver agreement and test–retest reliability were assessed using Gwet’s agreement coefficient (Gwet’s AC1) for EQ-5D dimensions and the intraclass correlation coefficient (ICC) for the EQ VAS.

**Results:**

Ninety-six patient-caregiver dyads participated in the study. Patient-caregiver agreement on the EQ-5D-Y descriptive system was moderate to good for both the Y-3L and Y-5L, but poor on the EQ VAS. Test–retest reliability of the descriptive system was good to very good for the Y-3L and moderate to good for the Y-5L in children, and fair to good for both versions of EQ-5D-Y in proxies. The EQ VAS showed good test–retest reliability in both children and caregivers. In a subgroup analysis of results in younger patients aged 8–10 years, patient-caregiver agreement and test–retest reliability were also observed to range from moderate to very good.

**Conclusion:**

Both the Y-3L and Y-5L descriptive systems showed acceptable patient-caregiver agreement and test–retest reliability when used to assess the HRQoL of children and adolescents with haematological malignancies, including in younger patients.

## Introduction

The 3-level EQ-5D (EQ-5D-3L) is a widely used measure of health status [1–4]. It was developed by the EuroQol Group in the 1980s as a brief, generic instrument to assess and value health outcomes in different populations [5]. In 2009, the EQ-5D-Y was designed as a version of EQ-5D which would be more suitable for use in respondents aged 8–15 years [6]. As a ‘youth’ version, the EQ-5D-Y retains the same five-dimension, three-level format of the EQ-5D-3L [7, 8], but is written in more appropriate language for children and adolescents. For example, the ‘anxiety/depression’ dimension in EQ-3D-3L was rephrased as ‘feeling worried, sad or unhappy’ in EQ-5D-Y. One advantage of having youth and adult versions of EQ-5D with similar content is that they can be useful in investigating the impact of childhood chronic conditions that last into adult life. The EQ-5D-Y was intended for use in a variety of settings, including clinical trials, population studies, and routine outcome measurement; moreover, when a value set becomes available, the EQ-5D-Y can be used as a preference-based instrument for quantifying quality-adjusted life-years in cost-utility analysis [9].

In 2019, a 5-level version of EQ-5D-Y (Y-5L) was developed by increasing the number of descriptive levels to five for each health dimension [10]. As with any new health-related quality-of-life (HRQoL) instrument, once developed, it is important to thoroughly test its psychometric properties in terms of its reliability, validity, and sensitivity. When measuring HRQoL in children, caregivers such as parents may have to serve as the proxy for children with poor literacy or whose health makes it impracticable for them to complete the questionnaire themselves. However, proxies may over- or under-estimate a child’s HRQoL so it is important to also assess the level of agreement between self- and proxy reports of HRQoL, especially if there is a need to compare or aggregate results from the two types of respondents. Assessment of proxy-children agreement has been performed in some studies for the Y-3L [11–14] but not for the Y-5L.

Test–retest reliability assesses another form of agreement, not between different raters as in the case of patient-caregiver agreement, but between the same rater on two different occasions [15]. It is also an important part of assessing an instrument’s measurement performance as it provides an indication of the amount of random error there may be in instrument scores. The test–retest reliability of the standard Y-3L has been assessed and demonstrated in general young populations [16] and paediatric patients with chronic kidney disease [17], but not in those with haematological malignancies. To date, there have been very few studies on the test–retest reliability of the Y-5L [18, 19].

This study aimed to simultaneously assess the patient-caregiver agreement and test–retest reliability of the self-complete and proxy versions of the 3- and 5-level variants of EQ-5D-Y (hereafter referred to as the Y-3Ls, Y-5Ls, and Y-3Lp, and Y-5Lp, respectively) in Chinese children and adolescents with haematological malignancies. The primary objectives of the present analysis were to: (1) examine and compare patient-caregiver agreement on the Y-3L and Y-5L and (2) assess the test–retest reliability of the self-complete and proxy versions of the Y-3L and Y-5L. A secondary aim was to assess these properties of the Y-3L and Y-5L questionnaires in a subgroup of patients aged 8–10 years, as younger children are sometimes considered less reliable respondents.

## Methods

### Sampling

Paediatric inpatients with leukaemia or other haematological malignancies and their caregivers were recruited from Shanghai Children Medical Centre from November 2018 to August 2019. All patients admitted to the wards for leukaemia or other haematological malignancies were invited to participate in the study. The inclusion criteria for the patients were: (1) a diagnosis of leukaemia or other haematological malignancy; (2) aged 8–17 years; (3) ability to converse in Chinese; (4) ability to understand questionnaires (based on a trained interviewer’s judgement). Children and adolescents who were not well enough for interview, who failed to cooperate due to cognitive impairment or mental disorders, did not give assent or whose legal guardians did not give consent were excluded. The inclusion criteria for caregivers were: (1) adult family member of an eligible patient; (2) being with the patient in the ward on the day of the survey; (3) ability to converse in Chinese; and (4) informed consent. Caregivers who were unwilling to participate or who were cognitively unable to complete the task were excluded. The study was approved by the institutional review board of Shanghai Jiaotong University (Project Identification Code: 2018087).

### Procedures

All consenting patient-caregiver dyads were interviewed in the haematology wards by a trained interviewer. All interviews were conducted in two parts. Caregivers first completed a baseline questionnaire which included: (1) questions on the patient’s socio-demographic characteristics including birth date, gender, education level, and disease duration; (2) the proxy version 1 of the Y-5L questionnaire; (3) the proxy version of the self-rated health (SRH) question for assessing the patient; (4) questions on the caregiver’s socio-demographic characteristics including relationship to patient, age, gender, educational attainment, monthly household income, and residential area, and; (5) the proxy version 1 of the Y-3L questionnaire (without the EQ-VAS). Proxy version 1 of the EQ-5D-Y asks the proxy to provide their own impression of the patient’s health on the day of the interview via the EQ-5D-Y descriptive system and the EQ VAS, in contrast to proxy version 2 which asks the proxy to try to imagine how the patient would rate their own health [6]. The paediatric patients then completed the second part of the questionnaire which included: (1) the beta version of the Y-5L questionnaire; (2) the same SRH question; and (3) the Y-3L questionnaire (without the EQ VAS, as this was already included in the Y-5L). All participants were invited to a face-to-face interview again in the same wards 2–13 days after the baseline interview. The structure of the follow-up interviews was the same as the baseline interviews except that the order of the Y-5L and Y-3L questionnaires was swapped for both patients and their caregivers to reduce any possible memory effect in the second visit.

On the days of the baseline and follow-up interviews, the patients were assessed by the interviewer using the Eastern Cooperative Oncology Group (ECOG) performance scale [20]. The interviewer also assessed the clinical characteristics of the patients, including mental consciousness and reactions, which can reflect the disease severity of patients and their ability to complete the interviews.

### Instruments

Both the Y-3L and Y-5L questionnaires consist of a five-dimension health-status descriptive system and a visual analogue scale (EQ VAS) on which respondents score their overall health on the day of the survey. The five dimensions comprising the descriptive system are: mobility, looking after myself, doing usual activities, having pain or discomfort, and feeling worried, sad or unhappy. Each dimension in Y-3L has three response options corresponding to the severity levels of no problems, some problems, and a lot of problems. Each dimension in Y-5L has five response options, corresponding to the levels of no problems, a little bit of a problem, some problems, a lot of problems, and extreme problems/cannot [8]. The expanded system aims to improve the ability of the Y-3L to discriminate among different levels of health and reduce any Y-3L ceiling effects [9]. The EQ VAS is an integral part of the EQ-5D-Y instrument and consists of a vertical, hash-marked numerical scale anchored by 0 (the worst imaginable health) at the bottom and 100 (the best imaginable health) on the top. An identical version of the EQ VAS is used in both versions of EQ-5D-Y.

The Y-3L questionnaire used in this study was the official, EuroQol-approved Chinese (for China) version while the Y-5L was translated by the investigators from the English version following the standard EuroQol Group translation guidelines [21]. The proxy and self-complete versions of the Y-5L are currently considered ‘beta’ versions by the EuroQol Group, i.e. they are undergoing psychometric testing before being considered for approval as official versions.

The self-rated health (SRH) question has been shown to be a valid measure of subjective health in instrument in children and adolescents [22]. The question in the present study was framed ‘How is your overall health today? Is it excellent, good, fair, poor or very poor’? The proxy version of SRH asked caregivers to rate their child’s health using the same 5-point response scale.

The ECOG (Eastern Cooperative Oncology Group) performance scale defines five different categories of performance status: 0 (fully active, no performance restriction); 1 (restricted in physically strenuous activity but ambulatory, able to carry out work of a light or sedentary nature); 2 (ambulatory and capable of all self-care but unable to carry out any work activities. Up and about more than 50% of waking hours); 3 (capable of only limited self-care, confined to bed or chair more than 50% of waking hours); 4 (completely disabled, cannot carry out any self-care. Totally confined to bed or chair) or 5 (dead) [20].

### Statistical analysis

The patient-caregiver agreement on the Y-3L and Y-5L at baseline was assessed using data from all patient-caregiver dyads. Patient-caregiver agreement on the EQ-5D-Y dimensions was assessed using Gwet’s agreement coefficient (Gwet’s AC1) [23]. A Gwet’s AC1 of < 0.2 was interpreted as poor agreement; 0.21–0.4 as fair; 0.41–0.6 as moderate; 0.61–0.8 as good and > 0.8 as very good [24]. Patient-caregiver agreement of the EQ VAS was assessed using the intraclass correlation coefficient (ICC). An ICC > 0.7 was considered to indicate good reliability [25].

In children, the test–retest reliability of the two versions of EQ-5D-Y was analysed using data from patients whose SRH remained unchanged between baseline and follow-up. Test–retest reliability of the proxy versions was assessed using data from patients whose health status was rated as unchanged by the same caregivers. Test–retest reliability for the five EQ-5D-Y dimensions was assessed using Gwet’s AC1 and that of the EQ VAS using ICC.

Lastly, subgroup analysis was performed to assess the patient-caregiver agreement and test–retest reliability of the EQ-5D-Y questionnaire in patients aged 8–10 years, as reliability is sometimes considered to be more difficult to achieve in younger respondents.

IBM SPSS version 25 (IBM Corp. Armonk, NewYork, USA) [26] and AgreeStat (version 2015.6, for Gwet’s AC1) was used to conduct all the analyses.

## Results

A total of 115 paediatric inpatients and their caregivers were invited to participate. Of those, 96 patient-caregiver dyads completed the baseline interviews, while 19 (16.5%) patients or their caregivers declined to complete the survey, primarily because they were worried that taking part in the interviews might worsen their child’s health. One caregiver, a grandmother, was excluded because she could not understand the questionnaires. Eighty-four (87.5%) of the dyads who participated at baseline also completed the follow-up interviews. Of the remainder, eight patients were discharged from hospital before follow-up, and four caregivers declined to participate in follow-up interviews. The mean (SD) time between responses to the first and second surveys was 2.8 (1.4) days (range 2–13 days). There were no missing responses on the descriptive system or the EQ VAS on any of the four versions of EQ-5D-Y tested in this study, either at baseline or follow-up.

The characteristics of the participants are shown in Table 1. The mean (SD) age of the 96 paediatric patients was 10.5 (2.2) years (range 8–17 years). The majority were boys (64.6%) and most had an ECOG performance score of 1 (56.3%). The most common diagnosis was acute lymphoblastic leukaemia (47.9%). Mean (SD) disease duration was 14.6 (18.8) months. The mean (SD) age of the 96 caregivers who completed the baseline interviews was 40.1 (9.3) years; 67.7% were mothers; 73.8% of the follow-up interviewers were completed by the same caregiver. The characteristics of the 84 dyads who also completed the follow-up interviews were similar to those who completed the baseline interviews (Table 1).

**Table 1:** Characteristics of patients and their caregivers

Characteristic	% (*n*)	
	Baseline (*n* = 96)	Follow-up (*n* = 84)
Child participants		
Age (year), mean (SD)	10.5 (2.2)	10.7 (2.2)
8–10	54.2 (52)	50.0 (42)
11–17	45.8 (44)	50.0 (42)
Sex		
Boys	64.6 (62)	60.7 (51)
Girls	35.4 (34)	39.3 (33)
Disease duration (month), mean (SD)	14.6 (18.8)	11.9 (17.8)
Diagnosis		
Acute lymphoblastic leukaemia	47.9 (46)	47.6 (40)
Non-Hodgkin’s lymphoma	26.0 (25)	26.2 (22)
Acute myeloid leukaemia	11.5 (11)	13.1 (11)
Rhabdomyosarcoma	4.2 (4)	3.6 (3)
Osteosarcoma	3.1 (3)	2.4 (2)
Other haematological malignancies	7.3(7)	7.1 (6)
ECOG		
0	16.7 (16)	19.0 (16)
1	56.3 (54)	53.6 (45)
2	20.8 (20)	21.4 (18)
3	6.3 (6)	6.0 (5)
SRH		
Excellent	21.8 (21)	20.2(17)
Good	35.4 (34)	35.7 (30)
Fair	41.7 (40)	42.9 (36)
Poor	1.0 (1)	1.2 (1)
Very poor	0.0 (0)	0.0 (0)
SRH (caregiver reported)		
Excellent	13.5 (13)	13.1 (11)
Good	37.5 (36)	44.0 (37)
Fair	41.7 (40)	36.9 (31)
Poor	6.3 (6)	4.8 (4)
Very poor	1.0 (1)	1.2 (1)
Caregivers		
Age (year), mean (SD)	40.1 (9.3)	39.0 (8.0)
Relationship to patient		
Father	20.8 (20)	26.2 (22)
Mother	67.7 (65)	61.9 (52)
Other	11.5 (11)	11.9 (10)

The performance scale defines five different performance statuses: 0 as fully active and no performance restrictions; 1 as strenuous physical activity restricted, fully ambulatory and able to carry out light work; 2 as capable of all looking after myself but unable to carry out any work activities. Up and about > 50% of waking hours; 3 as capable of only limited self-care, confined to bed or chair > 50% of waking hours; 4 as completely disabled; cannot carry out any self-care, totally confined to bed or chair; 5 as death

*ECOG* Eastern Cooperative Oncology Group, *SRH* the self-rated health

The baseline health status of the patients as described by the four EQ-5D-Y questionnaires is shown in Table 2. In each dimension, over half of the patients reported no problems and approximately one in five patients had no problems in all of the five dimensions. A slightly greater proportion of patients reported problems on the Y-5Ls compared to Y-3Ls, particularly in the ‘mobility’ dimension. Similar differences were observed between Y-3Lp and Y-5Lp (Table 2). Overall, on the descriptive system, proxies tended to rate patients’ health slightly better than the patients in all dimensions except for ‘feeling worried/sad/unhappy’. The mean proxy EQ VAS score (81.2; SD = 14.1) was lower than that based on the patients’ own assessment (85.8; SD = 15.1) by 4.6 points (*p* = 0.013).

**Table 2:** Baseline health status of patients measured by the four variants of the EQ-5D-Y questionnaire (*n* = 96)

Health dimension	% (*n*)					
	Level	Y-3Ls	Y-3Lp	Level	Y-5Ls	Y-5Lp
Mobility	No problems	65.6 (63)	74.0 (71)	No problems	60.4 (58)	67.7 (65)
	Some problems	33.3 (32)	20.8 (20)	A little bit of problems	30.2 (29)	22.9 (22)
	A lot of problems	1.0 (1)	5.2 (5)	Some problems	6.3 (6)	4.2 (4)
				A lot of problems	2.1 (2)	2.1 (2)
				Cannot do	1.0 (1)	3.1 (3)
Looking after myself	No problems	51.0 (49)	62.5 (60)	No problems	52.1 (50)	54.2 (52)
	Some problems	41.7 (40)	32.3 (31)	A little bit of problems	30.2 (29)	32.3 (31)
	A lot of problems	7.3 (7)	5.2 (5)	Some problems	10.4 (10)	8.3 (8)
				A lot of problems	0.0 (0)	2.1 (2)
				Cannot do	7.3 (7)	3.1 (3)
Doing usual activities	No problems	61.4 (59)	69.8 (67)	No problems	59.4 (57)	70.8 (68)
	Some problems	34.4 (33)	25.0 (24)	A little bit of problems	24.0 (23)	14.6 (14)
	A lot of problems	4.2 (4)	5.2 (5)	Some problems	11.4 (11)	7.3 (7)
				A lot of problems	1.0 (1)	3.1 (3)
				Cannot do	4.2 (4)	4.2 (4)
Having pain/discomfort	None	56.3 (54)	54.2 (52)	None	54.2 (52)	56.2 (54)
	Some	42.7 (41)	45.8 (44)	A little bit	32.3 (31)	36.4 (35)
	A lot	1.0 (1)	0.0 (0)	Some	11.4 (11)	6.2 (6)
				A lot	2.1 (2)	1.0 (1)
				Extreme	0.0 (0)	0.0 (0)
Feeling worried/sad/unhappy	No problems	60.4 (58)	53.1 (51)	No problems	60.4 (58)	51.0 (49)
	Some problems	38.5 (37)	41.7 (40)	A little bit of problems	27.1 (26)	34.4 (33)
	A lot of problems	1.0 (1)	5.2 (5)	Some problems	10.4 (10)	9.4 (9)
				A lot of problems	2.1 (2)	5.2 (5)
				Extreme problems	0.0 (0)	0.0 (0)
No problems in all dimensions		21.9 (21)	24.0 (23)		20.8 (20)	21.9 (21)

*Y-3Ls* self-complete version of the 3-level EQ-5D for youth, *Y-5Ls* self-complete version of the 5-level EQ-5D for youth, *Y-3Lp* proxy version of the 3-level EQ-5D for youth, *Y-5Lp* proxy version of the 5-level EQ-5D for youth

Patient-caregiver agreement on the EQ-5D dimensions is presented in Table 3. At baseline, Gwet’s AC1 ranged from 0.509 for ‘feeling worried/sad/unhappy’ to 0.708 for ‘having pain/discomfort’ for Y-3L, and from 0.561 for ‘feeling worried/sad/unhappy’ to 0.701 for ‘mobility’ for Y-5L. At follow-up, Gwet’s AC1 ranged from 0.563 for ‘having pain/discomfort’ to 0.769 for ‘looking after myself’ for Y-3L and from 0.503 for ‘doing usual activities’ to 0.629 for ‘looking after myself’ for Y-5L. The ICC value for the correlation between child and caregiver scores on the EQ VAS was 0.252 and 0.556 at baseline and follow-up, respectively.

**Table 3:** Patient-caregiver agreement on EQ-5D-Y dimensions at baseline and follow-up

Version	Dimension	Baseline (*n* = 96)		Follow-up (*n* = 84)	
		Gwet’s AC1 (95% CI)	Agreement (%)	Gwet’s AC1 (95% CI)	Agreement (%)
Y-3L	Mobility	0.653 (0.528, 0.781)	72.9	0.675 (0.546, 0.798)	75.0
	Looking after myself	0.587 (0.454, 0.717)	69.8	0.769 (0.658, 0.876)	83.3
	Doing usual activities	0.603 (0.466, 0.731)	69.8	0.644 (0.514, 0.781)	73.8
	Having pain/discomfort	0.708 (0.603, 0.822)	78.1	0.563 (0.421, 0.703)	67.8
	Feeling worried/sad/unhappy	0.509 (0.374, 0.639)	63.5	0.680 (0.555, 0.799)	76.2
Y-5L	Mobility	0.701 (0.603, 0.808)	74.0	0.582 (0.538, 0.750)	64.3
	Looking after myself	0.607 (0.487, 0.724)	66.7	0.629 (0.505, 0.751)	69.0
	Doing usual activities	0.628 (0.506, 0.736)	67.7	0.503 (0.37, 0.63)	58.3
	Having pain/discomfort	0.599 (0.478, 0.710)	65.6	0.603 (0.483, 0.733)	66.7
	Feeling worried/sad/unhappy	0.561 (0.440, 0.682)	62.5	0.565 (0.442, 0.688)	63.1

The test–retest reliability results for the five EQ-5D health dimensions are presented in Table 4. Using data from the 54 patients whose SRH remained unchanged from baseline to follow-up interviews, the Gwet’s AC1 values ranged from 0.628 for ‘having pain/discomfort’ to 0.901 for ‘doing usual activities’ for Y-3Ls, and from 0.562 for ‘having pain/discomfort’ to 0.678 for ‘mobility’ for Y-5Ls. Reliability for the proxy versions was calculated using data from 37 patients whose health status was rated as unchanged by the same caregiver using the SRH question. Gwet’s AC1 ranged from 0.267 (Y-3Lp) for ‘having pain/discomfort’ and 0.332 (Y-5Lp) for ‘mobility’ to 0.753 (Y-3Lp) and 0.688 (Y-5Lp) for ‘doing usual activities’, respectively. Using the same subsamples, the ICC value was 0.818 for the self-complete EQ VAS and 0.758 for the proxy version of EQ VAS.

**Table 4:** Test–retest reliability of EQ-5D-Y dimensions

Version	Dimension	Gwet’s AC1	95% CI	Agreement (%)
Y-3Ls	Mobility	0.689	0.533, 0.846	75.9
	Looking after myself	0.722	0.568, 0.876	79.6
	Doing usual activities	0.901	0.804, 0.997	92.6
	Having pain/discomfort	0.628	0.463, 0.793	72.2
	Feeling worried/sad/unhappy	0.685	0.530, 0.840	75.9
Y-5Ls	Mobility	0.678	0.532, 0.824	72.2
	Looking after myself	0.675	0.527, 0.822	72.2
	Doing usual activities	0.584	0.426, 0.743	64.8
	Having pain/discomfort	0.562	0.404, 0.721	63.0
	Feeling worried/sad/unhappy	0.658	0.511, 0.806	70.4
Y-3Lp	Mobility	0.590	0.376, 0.804	67.6
	Looking after myself	0.746	0.564, 0.927	81.1
	Doing usual activities	0.753	0.575, 0.931	81.1
	Pain/discomfort	0.267	0.037, 0.497	46.0
	Feeling worried/sad/unhappy	0.421	0.195, 0.647	56.8
Y-5Lp	Mobility	0.332	0.133, 0.533	43.2
	Looking after myself	0.624	0.435, 0.813	67.6
	Doing usual activities	0.688	0.513, 0.863	73.0
	Having pain/discomfort	0.343	0.149, 0.538	43.2
	Feeling worried/sad/unhappy	0.428	0.227, 0.629	51.4

The Gwet’s AC1 values were calculated using data from a subgroup of 54 and 37 patients whose health status was rated as unchanged by patients and caregivers, respectively

*Y-3Ls* self-complete version of the 3-level EQ-5D for youth, *Y-5Ls* self-complete version of the 5-level EQ-5D for youth, *Y-3Lp* proxy version of the 3-level EQ-5D for youth, *Y-5Lp* proxy version of the 5-level EQ-5D for youth

Results on the patient-caregiver agreement and test–retest reliability using data from patients aged 8–10 years were similar to those based on the entire sample (Tables 5, 6, 7 in “Appendix”). For example, regarding the test–retest reliability, the Gwet’s AC1 values ranged from 0.550 to 0.943 for Y-3Ls and from 0.495 to 0.750 for Y-5Ls (Table 6 in “Appendix”); the ICC value for the EQ VAS is 0.833.

## Discussion

This is the first study to perform an in-depth analysis of the patient-caregiver agreement and test–retest reliability of both the EQ-5D-Y-3L and the newly developed EQ-5D-Y-5L. Children and adolescents with haematological malignancies were considered a suitable population to assess the new version of EQ-5D-Y, due to the relatively high levels of morbidity and because the HRQoL of children and adolescents with haematological malignancies is affected not only by the disease itself but also by the side effects of radiation and chemotherapy [27]. It was considered that these characteristics would give a good spread of scores across dimensions and levels, which is important when assessing inter- and intra-rater reliability.

In general, we observed acceptable levels of agreement between children and adolescents with haematological malignancies and their caregivers using both the Y-3L or Y-5L. The same may not be said for the EQ VAS especially when children and adolescents and their caregivers have never used it before. The test–retest reliability of the Y-3L and Y-5L in children was also generally satisfactory and slightly better than that observed when using the proxy version in caregivers.

On the other hand, patient-caregiver agreement for the ‘mobility’, ‘looking after myself’ and ‘doing usual activities’ dimensions in our study was lower than that observed in previous studies [11, 12]. This could be due to the poor health of child participants in our study in those dimensions. When subjects are not very healthy and ceiling effects are low, variability in responses is expected to be greater, and thus observed reliability may be lower. The poor patient-caregiver agreement of the baseline EQ VAS scores in our study is in line with a Spanish study of 62 children with cerebral palsy and their parents where the ICC for EQ VAS was 0.581 (child–father) and 0.389 (child–mother) [14]. Interestingly, children and adolescents in our study reported higher EQ VAS scores than the caregivers while children in the Spanish study reported lower EQ VAS scores than their parents. It is possible that multiple factors, such as children’s ability and ways to interpret or use the EQ VAS and adaptation to illness, affect patient-caregiver agreement but the effects differ with the condition the children have and the culture they come from. It is not surprising that the patient-caregiver agreement in the EQ VAS was much poorer than the patient-caregiver agreement in the five health dimensions. This is because the EQ VAS is much more abstract and cognitively more difficult [28]. It is also possible that caregivers and children take different aspects of the child’s health into account when assigning a score on the VAS. Nevertheless, the patient-caregiver agreement of the EQ VAS improved considerably at follow-up in our study, which may suggest that greater agreement could be achieved once children and adolescents and their caregivers become familiar with the scale. However, the reasons for this improvement are unclear and warrant further investigation.

The test–retest reliability of both self-complete and proxy versions of Y-3L/Y-5L in this study was lower than that reported in previous studies [12, 16, 18, 29]. For example, in a Hong Kong study of 70 paediatric patients with idiopathic scoliosis, the Gwet’s AC1 ranged from 0.808 (having pain/discomfort) to 0.937 (looking after myself) for self-complete Y-5L [18]. A large proportion of children and adolescents in those studies reported ‘no problems’ with the EQ-5D dimensions, however, which could be the reason for the better reliability results. The moderate to good test–retest reliability of the self-complete EQ VAS in our study was similar to previous studies in Italy (ICC = 0.82) [16] and Spain (ICC = 0.855) [12], and higher than that in Japan (ICC = 0.40) [11] and Taiwan (ICC = 0.47) [17]. One of the reasons for the variations in the test–retest reliability results could be due to the varying test–retest intervals. Shorter intervals may result in memory effect during the completion of the questionnaires in the second interview, which could lead to better test–retest reliability results.

It is reassuring that both patient-caregiver agreement and test–retest reliability do not appear to be affected by the age of the children. Our results suggest that, by the age of 8 years, children can provide a reliable assessment of their own health using either version of the EQ-5D-Y, though it should be remembered that the questionnaire was administered in face-to-face interviews, and that results may not be equivalent to a situation in which the questionnaire was self-administered. The reliability of the EQ-5D-Y questionnaires in children aged 8–10 years in our study was higher than that of a study in Japan [11], in which reliability improved with age.

In this study, results on patient-caregiver agreement do not differ between the Y-3L and Y-5L. The magnitude of ceiling effects of Y-3L was only slightly greater compared to Y-5L. This is disappointing because one of the reasons underlying development of the Y-5L was to reduce ceiling effects. However, it is not entirely surprising. Studies of patients with juvenile idiopathic scoliosis showed that the Y-5L had only slightly fewer ceiling effects than the Y-3L [18, 29]. Regarding test–retest reliability, results were slightly poorer for the Y-5L than the Y-3L, which is consistent with the aforementioned studies of the patients with juvenile idiopathic scoliosis [29]. These findings suggest that increasing the number of response options leads to slightly less stable results over time, possibly indicating more random error in the Y-5L. On the other hand, test–retest reliability in the present study was assessed in patients and proxies who reported no change between the two visits on the self-rated overall health question. The SRH question, however, only provides a relatively blunt form of assessment and it is possible that minor variations in health within one EQ-5D-Y dimension which would be picked up by the Y-5L would not be detected by the SRH question. It is also possible that some losses in test–retest reliability will be offset by gains in responsiveness with the Y-5L, but that will be the subject of another analysis.

Our study has several strengths. First, we simultaneously assessed four different versions of the child-friendly EQ-5D including both self-complete and proxy versions. Second, the study design whereby the same children completed both the Y-3L and the Y-5L thereby facilitating comparison. Third, the use of SRH to ensure only those reporting no change in health status between visits were included in the test–retest analysis.

This study also had several limitations. First, all participants were recruited from one hospital in Shanghai and all the child participants had haematological malignancies; our findings might not be generalizable to children and adolescents who live in other regions or who have other medical conditions. Second, patients and caregivers were not separated when they completed the questionnaires. It is possible that some of them consulted each other when they answered the EQ-5D questionnaires, although the interviewers instructed them not to do so. Third, because of the unavailability of official versions of the Y-5L for interviewer administration, we used the beta version of Y-5L for self-completion by patients and proxies. It is possible, although unlikely, that the wording of these versions will change before they become official EuroQol versions. Finally, we used interviewer administration in the present study, and the results may not be generalisable to self-complete versions.

In conclusion, our study suggested that both the intra- and inter-rater reliability of the Y-3L and Y-5L descriptive systems is acceptable when the instruments are used by children and adolescents with haematological malignancies and their caregivers to assess HRQoL. Despite reasonable patient-caregiver agreement on the descriptive system, we would nevertheless recommend caution when comparing patient and proxy reported EQ-5D-Y data. This is even more true of the EQ VAS. Future research in this area should investigate results when using self-completed, rather than interviewer-administered versions of the questionnaires.

## Data Availability

The data that support the findings of this study are openly available in ‘Mendeley’ at: http://dx.doi.org/10.17632/bdnrj3fsyx.1. http://dx.doi.org/10.17632/793w7k59c3.1.

## Appendix

See Tables 5, 6 and 7.

**Table 5:** Patient-caregiver agreement of EQ-5D-Y measures at baseline and follow-up in patients aged 8–10 years

	Baseline (*n* = 52)			Follow-up (*n* = 43)		
	Gwet’s AC1 (95% CI)	Agreement (%)	ICC (95% CI)	Gwet’s AC1 (95% CI)	Agreement (%)	ICC (95% CI)
Y-3L						
Mobility	0.711 (0.554, 0.868)	76.9		0.826 (0.688, 0.964)	86.0	
Looking after myself	0.556 (0.369, 0.774)	67.3		0.841 (0.704, 0.978)	88.4	
Doing usual activities	0.633 (0.461, 0.805)	71.2		0.784 (0.632, 0.937)	83.7	
Having pain/discomfort	0.643 (0.476, 0.810)	73.1		0.656 (0.474, 0.838)	74.4	
Feeling worried/sad/unhappy	0.427 (0.231, 0.622)	57.7		0.628 (0.441, 0.815)	72.1	
Y-5L						
Mobility	0.761 (0.629, 0.892)	78.9		0.654 (0.484, 0.825)	69.8	
Looking after myself	0.620 (0.462, 0.778)	67.3		0.751 (0.599, 0.904)	79.1	
Doing usual activities	0.719 (0.578, 0.859)	75.0		0.619 (0.445, 0.793)	67.4	
Having pain/discomfort	0.528 (0.363, 0.694)	59.6		0.671 (0.505, 0.836)	72.1	
Feeling worried/sad/unhappy	0.548 (0.386, 0.711)	61.5		0.593 (0.417, 0.767)	65.1	
EQ VAS			0.235 (-0.038, 0.476)			0.605 (0.375, 0.765)

**Table 6:** Test–retest reliability of EQ-5D-Y dimensions in patients aged 8–10 years (*n*-23)

	Gwet’s AC1	95% CI	Agreement (%)
Y-3Ls			
Mobility	0.550	0.274, 0.826	65.2
Looking after myself	0.702	0.446, 0.957	78.2
Doing usual activities	0.943	0.826, 1.000	95.6
Having pain/discomfort	0.592	0.318, 0.866	69.6
Feeling worried/sad/unhappy	0.603	0.338, 0.869	69.6
Y-5Ls			
Mobility	0.750	0.543, 0.958	78.3
Looking after myself	0.702	0.478, 0.926	73.9
Doing usual activities	0.547	0.288, 0.807	60.9
Having pain/discomfort	0.542	0.290, 0.794	60.9
Feeling worried/sad/unhappy	0.495	0.234, 0.756	56.5

*Y-3Ls* self-complete version of the 3-level EQ-5D for youth, *Y-5Ls* self-complete version of the 5-level EQ-5D for youth

**Table 7:** Test–retest reliability of the EQ VAS (*n* = 23)

	Mean (SD)		ICC	95% CI
	Baseline	Follow-up		
EQ VAS	83.5 (22.6)	82.3 (22.7)	0.833	0.646, 0.926

*ICC* intraclass correlation coefficient
